# Ammonium (*E*)-3-(4-hy­droxy-3-meth­oxy­phen­yl)prop-2-enoate monohydrate

**DOI:** 10.1107/S1600536810042777

**Published:** 2010-10-30

**Authors:** Li-Cai Zhu

**Affiliations:** a School of Chemistry and Environment, South China Normal University, Guangzhou 510631, People’s Republic of China

## Abstract

In structure of the title compound ammonium ferulate monohydrate, NH_4_^+^·C_10_H_9_O_4_^−^·H_2_O, O—H⋯O and N—H⋯O hydrogen bonds link the ammonium cations, ferulate anions and water mol­ecules into a three-dimensional array. The ferulate anion is approximately planar, with a maximum deviation of 0.307 (2) Å.

## Related literature

For the biological activity of ferulic acid, see: Hirabayashi *et al.* (1995 ▶); Liyama *et al.* (1994 ▶); Nomura *et al.* (2003 ▶); Ogiwara *et al.* (2002 ▶); Ou *et al.* (2003 ▶).
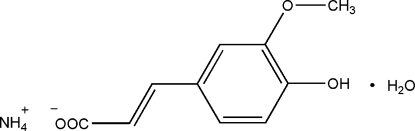


## Experimental

### 

#### Crystal data

NH_4_^+^·C_10_H_9_O_4_^−^·H_2_O
*M* *_r_* = 229.23
Monoclinic, 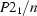

*a* = 8.6613 (19) Å
*b* = 8.3282 (18) Å
*c* = 16.457 (4) Å
β = 100.525 (3)°
*V* = 1167.1 (5) Å^3^
*Z* = 4
Mo *K*α radiation
μ = 0.11 mm^−1^
*T* = 296 K
0.30 × 0.27 × 0.26 mm

#### Data collection

Bruker APEXII diffractometer
5831 measured reflections
2090 independent reflections
1348 reflections with *I* > 2σ(*I*)
*R*_int_ = 0.040

#### Refinement

*R*[*F* ^2^ > 2σ(*F* ^2^)] = 0.043
*wR*(*F* ^2^) = 0.111
*S* = 1.01
2090 reflections
166 parameters
7 restraints
H atoms treated by a mixture of independent and constrained refinement
Δρ_max_ = 0.18 e Å^−3^
Δρ_min_ = −0.18 e Å^−3^

### 

Data collection: *APEX2* (Bruker, 2004 ▶); cell refinement: *SAINT* (Bruker, 2004 ▶); data reduction: *SAINT*; program(s) used to solve structure: *SHELXS97* (Sheldrick, 2008 ▶); program(s) used to refine structure: *SHELXL97* (Sheldrick, 2008 ▶); molecular graphics: *SHELXTL* (Sheldrick, 2008 ▶); software used to prepare material for publication: *SHELXL97*.

## Supplementary Material

Crystal structure: contains datablocks I, global. DOI: 10.1107/S1600536810042777/gk2311sup1.cif

Structure factors: contains datablocks I. DOI: 10.1107/S1600536810042777/gk2311Isup2.hkl

Additional supplementary materials:  crystallographic information; 3D view; checkCIF report

## Figures and Tables

**Table 1:** Hydrogen-bond geometry (Å, °)

*D*—H⋯*A*	*D*—H	H⋯*A*	*D*⋯*A*	*D*—H⋯*A*
O1*W*—H2*W*⋯O3^i^	0.86 (2)	2.07 (2)	2.918 (2)	167 (3)
O1*W*—H1*W*⋯O3	0.86 (2)	1.96 (2)	2.817 (2)	173 (3)
N1—H13⋯O4^ii^	0.90 (2)	2.06 (2)	2.904 (3)	156 (2)
N1—H12⋯O1*W*^i^	0.93 (2)	1.93 (2)	2.850 (3)	175 (2)
N1—H11⋯O1^iii^	0.93 (2)	2.25 (2)	3.043 (3)	144 (2)
N1—H11⋯O2^iii^	0.93 (2)	2.14 (2)	2.823 (2)	130 (2)
N1—H10⋯O4^iv^	0.94 (2)	1.83 (2)	2.761 (3)	169 (2)
O2—H2⋯O3^v^	0.82	1.81	2.594 (2)	160

Symmetry codes: (i) 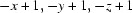; (ii) 
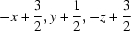
; (iii) 
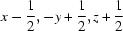
; (iv) 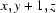; (v) 
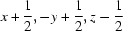
.

**Fig. 1.**
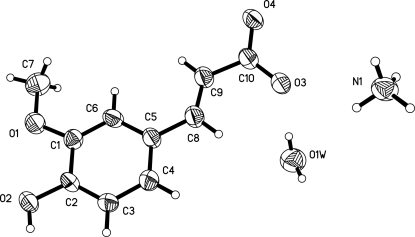
The molecular structure showing the atomic-numbering scheme. Displacement ellipsoids are drawn at the 50% probability level.

**Fig. 2.**
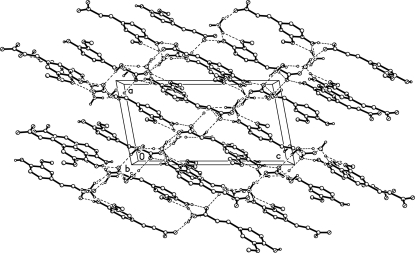
The crystal packing showing the hydrogen bonding interactions as broken lines.

## supplementary crystallographic information

### Comment

3-(4-Hydroxy-3-methoxyphenyl)-2-propenoic acid, also known as ferulic acid, is one of the main endogenous phenolic acids in plant kingdom (Liyama *et al.*, 1994). Attention was paid to the structural modifications of ferulic acid owing to its extensive bioactivities including anti-platelet aggregation, anti-oxidation, anti-inflammation, anti-tumor, anti-mutagenicity, antibiosis and immunity enchancement (Hirabayashi *et al.*, 1995; Ogiwara *et al.*, 2002). A series of ferulic acid derivatives were designed and synthesized, such as their salts, esters, ethers and amides, and some of them show the better bioactivities than those of ferulic acid (Nomura *et al.*, 2003; Ou *et al.*, 2003). The molecular and crystal structure of the title compound is presented in this article.

In the asymmetric unit of the title compound, illustrated in Fig. 1, there are an ammonium cation, one singly deprotonated 3-(4-hydroxy-3-methoxyphenyl)-2-propenoate anion, and one water molecule. The molecules are self-assembled by various O—H···O and N—H···O hydrogen bonds (Table 1 and Fig. 2), resulting in the formation of a three-dimensional supramolecular network.

### Experimental

A mixture of ferulic acid (0.388 g, 2 mmol) and ammonia (0.15 ml, 2 mmol) was stirred with methanol (20 ml) for 0.5 h at room temperature. After several days colourless block-like crystals, suitable for X-ray diffraction analysis, were obtained by slow evaporation of the solution.

### Refinement

The H atoms of water molecule and ammonium cation were found from difference Fourier maps and refined isotropically with a restraint of O—H = 0.87 (2) Å and H_1W_···H_2W_ = 1.39 (2) Å for water molecule, N—H = 0.87 (2) Å for ammonium cation, and *U*_iso_(H) = 1.5 *U*_eq_(O, N). All other H atoms were positioned geometrically and refined as riding, with C—H = 0.93–0.96 Å, O—H = 0.82 Å, and with *U*_iso_(H) = 1.2 or 1.5 *U*_eq_(C, O).

### Crystal data

**Table:**

NH_4_^+^·C_10_H_9_O_4_^−^·H_2_O	*F*(000) = 488
*M**_r_* = 229.23	*D*_x_ = 1.305 Mg m^−^^3^
Monoclinic, *P*2_1_/*n*	Mo *K*α radiation, λ = 0.71073 Å
Hall symbol: -P 2yn	Cell parameters from 1012 reflections
*a* = 8.6613 (19) Å	θ = 2.5–21.0°
*b* = 8.3282 (18) Å	µ = 0.11 mm^−^^1^
*c* = 16.457 (4) Å	*T* = 296 K
β = 100.525 (3)°	Block, colourless
*V* = 1167.1 (5) Å^3^	0.30 × 0.27 × 0.26 mm
*Z* = 4	

### Data collection

**Table:**

Bruker APEXII diffractometer	1348 reflections with *I* > 2σ(*I*)
Radiation source: fine-focus sealed tube	*R*_int_ = 0.040
graphite	θ_max_ = 25.2°, θ_min_ = 2.5°
φ and ω scan	*h* = −9→10
5831 measured reflections	*k* = −9→9
2090 independent reflections	*l* = −19→19

### Refinement

**Table:**

Refinement on *F*^2^	Secondary atom site location: difference Fourier map
Least-squares matrix: full	Hydrogen site location: inferred from neighbouring sites
*R*[*F*^2^ > 2σ(*F*^2^)] = 0.043	H atoms treated by a mixture of independent and constrained refinement
*wR*(*F*^2^) = 0.111	*w* = 1/[σ^2^(*F*_o_^2^) + (0.0482*P*)^2^ + 0.106*P*] where *P* = (*F*_o_^2^ + 2*F*_c_^2^)/3
*S* = 1.01	(Δ/σ)_max_ < 0.001
2090 reflections	Δρ_max_ = 0.18 e Å^−^^3^
166 parameters	Δρ_min_ = −0.18 e Å^−^^3^
7 restraints	Extinction correction: *SHELXL97* (Sheldrick, 2008), Fc^*^=kFc[1+0.001xFc^2^λ^3^/sin(2θ)]^-1/4^
Primary atom site location: structure-invariant direct methods	Extinction coefficient: 0.011 (2)

### Special details

**Table:**

Geometry. All e.s.d.'s (except the e.s.d. in the dihedral angle between two l.s. planes) are estimated using the full covariance matrix. The cell e.s.d.'s are taken into account individually in the estimation of e.s.d.'s in distances, angles and torsion angles; correlations between e.s.d.'s in cell parameters are only used when they are defined by crystal symmetry. An approximate (isotropic) treatment of cell e.s.d.'s is used for estimating e.s.d.'s involving l.s. planes.
Refinement. Refinement of *F*^2^ against ALL reflections. The weighted *R*-factor *wR* and goodness of fit *S* are based on *F*^2^, conventional *R*-factors *R* are based on *F*, with *F* set to zero for negative *F*^2^. The threshold expression of *F*^2^ > σ(*F*^2^) is used only for calculating *R*-factors(gt) *etc*. and is not relevant to the choice of reflections for refinement. *R*-factors based on *F*^2^ are statistically about twice as large as those based on *F*, and *R*- factors based on ALL data will be even larger.

### Fractional atomic coordinates and isotropic or equivalent isotropic displacement parameters (Å^2^)

**Table:**

	*x*	*y*	*z*	*U*_iso_*/*U*_eq_	
C1	0.9208 (2)	−0.0647 (2)	0.33845 (13)	0.0374 (5)	
C2	0.9382 (2)	0.0503 (2)	0.27793 (12)	0.0361 (5)	
C3	0.8765 (2)	0.2015 (2)	0.28226 (13)	0.0413 (5)	
H3	0.8884	0.2782	0.2428	0.050*	
C4	0.7969 (2)	0.2398 (3)	0.34519 (14)	0.0432 (6)	
H4	0.7547	0.3421	0.3470	0.052*	
C5	0.7786 (2)	0.1292 (2)	0.40548 (13)	0.0387 (5)	
C6	0.8419 (2)	−0.0247 (2)	0.40114 (13)	0.0402 (5)	
H6	0.8306	−0.1007	0.4410	0.048*	
C7	0.9605 (3)	−0.3375 (3)	0.38312 (16)	0.0617 (7)	
H7A	1.0047	−0.3081	0.4389	0.093*	
H7B	1.0094	−0.4342	0.3687	0.093*	
H7C	0.8496	−0.3550	0.3785	0.093*	
C8	0.6979 (2)	0.1798 (3)	0.47158 (13)	0.0438 (6)	
H8	0.6529	0.2815	0.4650	0.053*	
C9	0.6790 (2)	0.1034 (3)	0.53912 (13)	0.0438 (6)	
H9	0.7113	−0.0031	0.5454	0.053*	
C10	0.6093 (3)	0.1780 (3)	0.60560 (14)	0.0413 (5)	
O1	0.98669 (18)	−0.21136 (16)	0.32848 (9)	0.0492 (4)	
O2	1.01519 (18)	0.00231 (17)	0.21705 (9)	0.0479 (4)	
H2	1.0125	0.0744	0.1829	0.072*	
O3	0.5464 (2)	0.31626 (18)	0.59297 (9)	0.0557 (5)	
O4	0.61918 (18)	0.10581 (17)	0.67298 (9)	0.0503 (4)	
N1	0.6808 (3)	0.7821 (2)	0.69610 (14)	0.0506 (5)	
H10	0.674 (3)	0.894 (2)	0.6905 (15)	0.076*	
H11	0.595 (2)	0.740 (3)	0.7154 (16)	0.076*	
H12	0.685 (3)	0.733 (3)	0.6460 (12)	0.076*	
H13	0.764 (2)	0.748 (3)	0.7335 (14)	0.076*	
O1W	0.3143 (2)	0.3867 (2)	0.45407 (11)	0.0698 (6)	
H1W	0.381 (3)	0.358 (3)	0.4970 (14)	0.105*	
H2W	0.340 (3)	0.481 (2)	0.4400 (18)	0.105*	

### Atomic displacement parameters (Å^2^)

**Table:**

	*U*^11^	*U*^22^	*U*^33^	*U*^12^	*U*^13^	*U*^23^
C1	0.0408 (12)	0.0330 (12)	0.0394 (13)	−0.0007 (9)	0.0098 (10)	−0.0022 (9)
C2	0.0411 (12)	0.0403 (12)	0.0302 (12)	−0.0040 (10)	0.0150 (10)	−0.0019 (9)
C3	0.0522 (14)	0.0365 (12)	0.0379 (13)	0.0032 (10)	0.0157 (11)	0.0069 (10)
C4	0.0514 (14)	0.0374 (12)	0.0436 (14)	0.0050 (10)	0.0160 (11)	0.0022 (10)
C5	0.0458 (13)	0.0380 (12)	0.0351 (12)	0.0004 (10)	0.0146 (10)	−0.0029 (10)
C6	0.0506 (13)	0.0395 (12)	0.0325 (12)	−0.0025 (10)	0.0128 (10)	0.0036 (10)
C7	0.0783 (19)	0.0395 (14)	0.0704 (19)	0.0025 (12)	0.0220 (15)	0.0129 (12)
C8	0.0524 (14)	0.0392 (12)	0.0431 (14)	0.0014 (10)	0.0171 (11)	−0.0034 (10)
C9	0.0572 (14)	0.0362 (12)	0.0414 (14)	0.0010 (11)	0.0182 (11)	−0.0016 (10)
C10	0.0491 (14)	0.0399 (13)	0.0374 (13)	−0.0037 (11)	0.0143 (11)	−0.0042 (11)
O1	0.0673 (10)	0.0356 (9)	0.0503 (10)	0.0052 (7)	0.0253 (8)	0.0039 (7)
O2	0.0635 (10)	0.0432 (9)	0.0443 (10)	0.0073 (8)	0.0290 (8)	0.0043 (7)
O3	0.0868 (13)	0.0443 (10)	0.0416 (10)	0.0147 (8)	0.0259 (9)	0.0026 (7)
O4	0.0731 (11)	0.0449 (9)	0.0375 (9)	0.0043 (8)	0.0223 (8)	0.0025 (7)
N1	0.0660 (15)	0.0430 (12)	0.0436 (13)	−0.0077 (11)	0.0120 (11)	0.0037 (10)
O1W	0.0854 (14)	0.0635 (12)	0.0582 (13)	0.0016 (11)	0.0068 (10)	−0.0008 (10)

### Geometric parameters (Å, °)

**Table:**

C1—O1	1.371 (2)	C7—H7C	0.9600
C1—C6	1.378 (3)	C8—C9	1.317 (3)
C1—C2	1.409 (3)	C8—H8	0.9300
C2—O2	1.361 (2)	C9—C10	1.480 (3)
C2—C3	1.375 (3)	C9—H9	0.9300
C3—C4	1.383 (3)	C10—O4	1.250 (2)
C3—H3	0.9300	C10—O3	1.274 (2)
C4—C5	1.384 (3)	O2—H2	0.8200
C4—H4	0.9300	N1—H10	0.942 (17)
C5—C6	1.401 (3)	N1—H11	0.927 (17)
C5—C8	1.458 (3)	N1—H12	0.926 (17)
C6—H6	0.9300	N1—H13	0.903 (17)
C7—O1	1.428 (2)	O1W—H1W	0.863 (16)
C7—H7A	0.9600	O1W—H2W	0.863 (16)
C7—H7B	0.9600		
			
O1—C1—C6	125.42 (19)	O1—C7—H7C	109.5
O1—C1—C2	114.84 (18)	H7A—C7—H7C	109.5
C6—C1—C2	119.74 (19)	H7B—C7—H7C	109.5
O2—C2—C3	123.65 (18)	C9—C8—C5	129.8 (2)
O2—C2—C1	116.80 (18)	C9—C8—H8	115.1
C3—C2—C1	119.54 (18)	C5—C8—H8	115.1
C2—C3—C4	120.12 (19)	C8—C9—C10	123.5 (2)
C2—C3—H3	119.9	C8—C9—H9	118.3
C4—C3—H3	119.9	C10—C9—H9	118.3
C3—C4—C5	121.45 (19)	O4—C10—O3	122.48 (19)
C3—C4—H4	119.3	O4—C10—C9	118.9 (2)
C5—C4—H4	119.3	O3—C10—C9	118.6 (2)
C4—C5—C6	118.34 (19)	C1—O1—C7	117.54 (17)
C4—C5—C8	118.43 (19)	C2—O2—H2	109.5
C6—C5—C8	123.21 (19)	H10—N1—H11	111 (2)
C1—C6—C5	120.79 (19)	H10—N1—H12	111 (2)
C1—C6—H6	119.6	H11—N1—H12	108 (2)
C5—C6—H6	119.6	H10—N1—H13	114 (2)
O1—C7—H7A	109.5	H11—N1—H13	104 (2)
O1—C7—H7B	109.5	H12—N1—H13	108 (2)
H7A—C7—H7B	109.5	H1W—O1W—H2W	108 (2)

### Hydrogen-bond geometry (Å, °)

**Table:**

*D*—H···*A*	*D*—H	H···*A*	*D*···*A*	*D*—H···*A*
O1W—H2W···O3^i^	0.86 (2)	2.07 (2)	2.918 (2)	167 (3)
O1W—H1W···O3	0.86 (2)	1.96 (2)	2.817 (2)	173 (3)
N1—H13···O4^ii^	0.90 (2)	2.06 (2)	2.904 (3)	156 (2)
N1—H12···O1W^i^	0.93 (2)	1.93 (2)	2.850 (3)	175 (2)
N1—H11···O1^iii^	0.93 (2)	2.25 (2)	3.043 (3)	144 (2)
N1—H11···O2^iii^	0.93 (2)	2.14 (2)	2.823 (2)	130 (2)
N1—H10···O4^iv^	0.94 (2)	1.83 (2)	2.761 (3)	169 (2)
O2—H2···O3^v^	0.82	1.81	2.594 (2)	160

Symmetry codes: (i) −*x*+1, −*y*+1, −*z*+1; (ii) −*x*+3/2, *y*+1/2, −*z*+3/2; (iii) *x*−1/2, −*y*+1/2, *z*+1/2; (iv) *x*, *y*+1, *z*; (v) *x*+1/2, −*y*+1/2, *z*−1/2.
